# Innovative Technologies: Organic Solar Cells

**Published:** 2005-05

**Authors:** John Manuel

Photovoltaic cells that convert sunlight into electricity have been around for decades, yet their commercial use has been largely limited to applications where conventional electric power is difficult or impossible to provide, such as lighting of road signs and offshore buoys. The problem is primarily economic—although sunlight is free, the high cost of manufacturing traditional silicon-based solar cells has limited their penetration into markets where coal, nuclear, and other nonrenewable sources currently provide more economical energy. Researchers at the Georgia Institute of Technology have developed a new type of solar cell that may someday change that equation.

Bernard Kippelen, a professor in the Center for Organic Photonics and Electronics and the School of Electrical and Computer Engineering at Georgia Tech, is leading studies into the use of pentacene as a medium for converting sunlight to electricity. Pentacene, a compound of carbon and hydrogen, can form a crystalline film in which molecules assemble in an ordered pattern. This makes the compound more conducive to the flow of electricity than the disordered organic compounds that have been tested in the past for possible photovoltaic applications. Improved conductivity leads to higher efficiency, and if that quality can be combined with low cost of manufacture and ease of use, the material holds great promise.

In an article published in the 29 November 2004 issue of *Applied Physics Letters*, Kippelen and fellow research scientists Seunghyup Yoo and Benoit Domercq describe their tests of an organic film made of pentacene combined with a form of carbon known as C_60_. The organic layers and an electrode were sequentially deposited onto indium–tin oxide substrates. Broadband illumination was provided by a lamp, photocurrent was measured under varying light spectrums, and conversion efficiencies (the amount of light converted into electricity) were calculated.

The team was able to convert solar energy into electricity with 2.7% efficiency; in unpublished tests since then, they demonstrated power conversion efficiencies of 3.4%. Kippelen believes they will be able to reach 5% in the near future.

Commercial photovoltaic cells that employ silicon crystals are 12–15% efficient, but they are expensive to manufacture and run. Complete systems, including installation, produce electricity at a cost equivalent of 20–40¢ per kilowatt hour (depending upon scale of system and financing) versus 8–12¢ per kilowatt hour for electricity generated by conventional power plants. Kippelen says development of thin-film organic solar cells is not far enough along to estimate the costs of energy production. However, the thin-film cells could possibly be manufactured in a roll-to-toll process, significantly lowering their cost and narrowing the gap with fossil fuel–generated electricity.

Kippelen is confident that his product’s unique properties will allow it to be used in applications for which silicon cells are not appropriate. Whereas silicon cells are rigid and relatively thick at 100 microns across, thin-film organic solar cells are lightweight, flexible, and less than 1 micron thick. This could open up new markets for solar energy, perhaps powering small electronic devices such as radiofrequency identification tags, MP3 players, and laptop computers. Kippelen estimates that organic solar cells are at least five years away from residential applications but could find niche low-power applications within two years.

However, thin-film solar power will need to be deployed on a much larger scale if it is to significantly improve the environment. “Small electronic devices represent a miniscule part of total energy consumption,” says Tom Starrs, chairman of the American Solar Energy Society. “For any photovoltaic technology to make a significant contribution to global energy needs, it needs to be interconnected with the electrical grid, displacing power generated by coal, nuclear, and other nonrenewable sources of energy.”

## Figures and Tables

**Figure:**
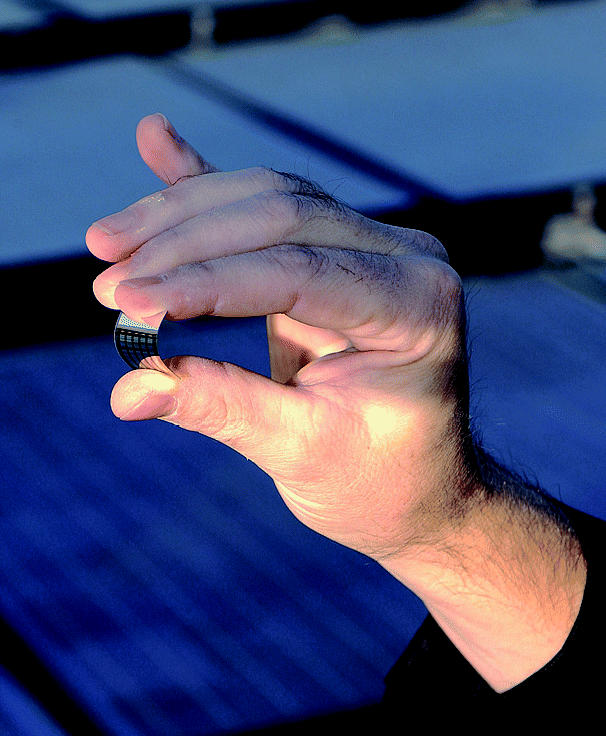
**Solar sensation.** New thin-film technology offers promise in converting solar energy into electricity.

